# CLEC4F Is an Inducible C-Type Lectin in F4/80-Positive Cells and Is Involved in Alpha-Galactosylceramide Presentation in Liver

**DOI:** 10.1371/journal.pone.0065070

**Published:** 2013-06-06

**Authors:** Chih-Ya Yang, Jiun-Bo Chen, Ting-Fen Tsai, Yi-Chen Tsai, Ching-Yen Tsai, Pi-Hui Liang, Tsui-Ling Hsu, Chung-Yi Wu, Mihai G. Netea, Chi-Huey Wong, Shie-Liang Hsieh

**Affiliations:** 1 Institute of Microbiology and Immunology, National Yang-Ming University, Taipei, Taiwan; 2 Program in Molecular Medicine, National Yang-Ming University and Academia Sinica, Taipei, Taiwan; 3 Department of Life Sciences and Institute of Genome Sciences, National Yang-Ming University, Taipei, Taiwan; 4 Transgenic Core Facility, Institute of Molecular Biology, Academia Sinica, Taipei, Taiwan; 5 School of Pharmacy, College of Medicine, National Taiwan University, Taipei, Taiwan; 6 Genomics Research Center, Academia Sinica, Taipei, Taiwan; 7 Department of Internal Medicine, Radboud University Nijmegen Medical Centre, Nijmegen, Netherlands; 8 Institute of Clinical Medicine & Infection and Immunity Center, National Yang-Ming University, Taipei, Taiwan; 9 Immunology Center, Taipei Veterans General Hospital, Taipei, Taiwan; 10 The Institute for Cancer Biology and Drug Discovery, College of Medical Science and Technology, Taipei Medical University, Taipei, Taiwan; King’s College London School of Medicine, United Kingdom

## Abstract

CLEC4F, a member of C-type lectin, was first purified from rat liver extract with high binding affinity to fucose, galactose (Gal), N-acetylgalactosamine (GalNAc), and un-sialylated glucosphingolipids with GalNAc or Gal terminus. However, the biological functions of CLEC4F have not been elucidated. To address this question, we examined the expression and distribution of murine CLEC4F, determined its binding specificity by glycan array, and investigated its function using CLEC4F knockout (*Clec4f−/−*) mice. We found that CLEC4F is a heavily glycosylated membrane protein co-expressed with F4/80 on Kupffer cells. In contrast to F4/80, CLEC4F is detectable in fetal livers at embryonic day 11.5 (E11.5) but not in yolk sac, suggesting the expression of CLEC4F is induced as cells migrate from yolk cells to the liver. Even though CLEC4F is not detectable in tissues outside liver, both residential Kupffer cells and infiltrating mononuclear cells surrounding liver abscesses are CLEC4F-positive upon *Listeria monocytogenes* (*L. monocytogenes*) infection. While CLEC4F has strong binding to Gal and GalNAc, terminal fucosylation inhibits CLEC4F recognition to several glycans such as Fucosyl GM1, Globo H, Bb3∼4 and other fucosyl-glycans. Moreover, CLEC4F interacts with alpha-galactosylceramide (α-GalCer) in a calcium-dependent manner and participates in the presentation of α-GalCer to natural killer T (NKT) cells. This suggests that CLEC4F is a C-type lectin with diverse binding specificity expressed on residential Kupffer cells and infiltrating monocytes in the liver, and may play an important role to modulate glycolipids presentation on Kupffer cells.

## Introduction

CLEC4F (also known as Kupffer cell receptor, KCR) was first purified from rat liver extract by affinity chromatography on L-fucosyl-bovine serum albumin (BSA)-Sepharose column and was reported as a hepatic fucose-binding lectin. This suggested that rat CLEC4F (rCLEC4F) serves as fucose receptor [1], [2]. Further studies showed that rCLEC4F has high binding affinity for Gal and GalNAc [1], [3], desialylated, complex N-link glycan [4] and glycolipids, such as Gb4Cer (GalNAcβ1-3Galα1-4Galβ1-4GlcβlCer), Gb5Cer (GalNAcα1-3GalNAcβ1-3Galα1-4Galβ1-4GlcβlCer) and LacCer (Galβ1-4GlcβlCer) but not ganglinoside [5]. Based on the recognition patterns, rCLEC4F may have functions paralleling to mammalian CLEC4H/asialoglycoprotein receptor [4] and may also participate in the clearance of circulating Gal- and fucose-terminated glycoproteins from blood [4], [6].

The rCLEC4F is a 550 amino acid type II transmembrane C-type lectin [7] and the C-terminal domain of rCLEC4F is homologous to the carbohydrates recognition domain (CRD) of other mammalian C-type lectins, such as langerin, asialoglycoprotein receptor and collectin. The extracellular domain of rCLEC4F exists as a trimer stabilized by the neck region [4]. The expression of rCLEC4F was shown to be exclusively present on resident liver macrophages [2], thus rCLEC4F is also named as Kupffer cell receptor/KCR. Kupffer cells are heterogeneous and are the largest population of tissue macrophages located in the liver within the lumen of hepatic sinusoids [8], [9], [10]. F4/80 is the pan marker for murine tissue macrophages [11], [12], [13]. In general, murine Kupffer cells were identified by F4/80^+^ cells in the liver. However, there is no suitable marker to distinguish Kupffer cells from other tissue macrophages and the mononuclear phagocyte system. Hence, studies on the ontogeny of Kupffer cells are still controversial [9], [13]. Recent studies show that the mRNA expression level of CLEC4F was enriched in carbon-labeled Kupffer cells [14] and hepatic inflammation [15], but was decreased or lost in Kupffer cells-depleted livers [16], [17]. Nevertheless, the expression of CLEC4F in the mononuclear phagocyte system and tissues are not confirmed yet. Therefore, we would like to examine both the mRNA and protein level of CLEC4F to clarify the distribution and specificity of CLEC4F.

CLEC4F belongs to the C-type lectin family, but its biological functions are not well elucidated yet. It has been shown that members of the C-type lectin family are involved in the delivery of lipid antigens to antigen presenting cells. The lipoarabinomannan has been shown to be internalized and delivered to late endosomal and lysosomal compartments by the CLEC13D/CD206/macrophage mannose receptor [18], while the presentation of CD1a-dependent lipid antigens is blocked by antibodies to CLEC4K/CD207/langerin [19]. NKT cells are a unique lineage of T cells that express NK lineage receptors and semi-invariant CD1d-restriced αβ TCRs [20], [21] and can be activated by glycolipid α-GalCer [22]. Upon stimulation, NKT cells produce large amounts of IL-4, IFN-γ and other cytokines, potentially enabling them to act as powerful regulators of the immune system [23]. Mouse NKT cells represent about 0.5% of the T cell population in blood, peripheral lymph node, and spleen. Almost 30% of the T cells in the liver have the NKT cell phenotype [24]. It has been shown that Kupffer cells are the key antigen presentation cells for hepatic NKT cells [25]. However, the role of Kupffer cells in glycolipid presentation and NKT activation is still controversial. During *Borrelia burgdorferi* infection, Kupffer cells form stable contacts with NKT cells via CD1d, which lead NKT cells activation [26]. In contrast, other studies have shown Kupffer cells were not required in the development of α-GalCer-induced liver injury [27] or α-GalCer-induced NKT cell hyporesponsiveness [28]. Therefore, we are interested in understanding whether CLEC4F is involved in presentation of α-GalCer.

In order to investigate these issues, anti-CLEC4F monoclonal antibodies (mAbs) and *Clec4f−/−* mice were generated to determine the expression and distribution of CLEC4F-positive (CLEC4F^+^) cells, and to understand the ontogeny of Kupffer cells. Moreover, mice were challenged with *L. monocytogenes* to examine the distribution of CLEC4F^+^ cells and investigate its function in host defense. Here we reported that CLEC4F is expressed on residential Kupffer cells and F4/80-positive cells infiltrating into liver. Furthermore, CLEC4F is involved in the presentation of α-GalCer to NKT cells, suggesting CLEC4F is not only a tissue macrophage-specific marker, but may also play a role in glycolipid presentation.

## Materials and Methods

### Animals

C57BL/6 mice were obtained from the National Laboratory Animal Center, Taiwan. *Clec4f−/−* mice were generated by homologous recombination in ES cells (AB2.2, 129/SvEv strain) (Figure S1 in File S1) and backcrossed onto C57BL/6 background for more than eight generations. To destroy the *clec4f* gene, the targeting vector was constructed to inset the enhanced green fluorescent protein (EGFP) gene into the exon 4 of *clec4f* gene to disrupt the expression of endogenous CLEC4F. Murine genomic DNA of *clec4f* was obtained by screening lambda phage library derived from mouse 129Sv/Ev strain. The 3.7 kb EcoRI DNA fragment, which contains exon 1, exon 2, exon 3 and partial exon 4 of *Clec4f* gene, and the 2.3 kb EcoRI/HindIII DNA fragment, which contains exon 5, were used for the left arm and right arm of targeting vector, respectively. The *Clec4f* targeting vector, which contains EGFP and neomycin selection cassette, was linearized with KpnI and transfected into AB2.2 ES cells by electroporation. Targeted ES cells were screened by Southern blot analysis using a specific 3′-flanking probe and then were injected into C57BL/6 blastocysts. Chimeric male mice were bred with C57BL/6 females. Germ-line transmission was obtained from their agouti progeny. All animal protocols were approved by the Institutional Animal Care and Use Committee (IACUC) in Taiwan. All mice were bred and housed at the laboratory animal center, and the procedures were approved by the Institutional Animal Care and usage committee of National Yang-Ming University.

### Cloning, Expression and Purification of Murine CLEC4F

The full-length of murine CLEC4F was cloned by reverse-transcription polymerase chain reaction (RT-PCR) using BALB/c liver cDNA as templates (forward primer: 5′-AAGGAGGCGGAACTGAACA-3′; reverse primer: 5′-CTAGCCTACTCTGGCCGC-3′) and subcloned into pFLAG-CMV2 vector (Kodak Co.). The expression of full length CLEC4F was expressed by transfecting the pFLAG-CMV2-CLEC4F into 293T cells and confirmed by Western blot using anti-FLAG M2 mAb (Sigma-Aldrich). The extracellular domain of CLEC4F was cloned by similar methods, except the forward primer was replaced with 5′-CTAGCCTACTCTGGCCGC-3′. The cDNA fragment of CLEC4F ECD was subcloned into pcDNA/3hIgG1.Fc to generate C-terminal hIgG1 Fc-tagged fusion proteins. The recombinant Fc.CLEC4F fusion protein was overexpressed using the FreeStyle 293 Expression System (Invitrogen) and the recombinant Fc.CLEC4F fusion protein was purified by Protein A columns (GE Healthcare) [29].

### Generation and Screen of Anti-CLEC4F mAbs

BALB/c mice were immunized with purified recombinant CLEC4F protein as antigen mixed with complete Freund’s adjuvant (Sigma-Aldrich). The suitable mouse with highest titer was selected for administration of the final boost. Total splenocytes were fused with mouse myeloma NS-1 cells in the presence of 50% (v/v) polyethylene glycol (PEG1450, Sigma-Aldrich). Fused cells were cultured in HAT selection medium and the medium was refreshed after one week. 2 weeks after fusion, culture supernatants were screened by ELISA to identify the candidate clones for further analysis by limiting dilution. Anti-CLEC4F mAbs were selected by ELISA-based differential screening, and only those recognizing recombinant CLEC4F were regarded as positive clones.

### RNA Isolation and Real-time Quantitative RT-PCR (qRT-PCR)

Total RNA was extracted from cells or tissues by using Trizol Reagent (Invitrogen). RNA (2 µg) was reverse transcribed in to complementary DNA (cDNA) by using oligo(dT) primers and Strata-Script™ RT-PCR kit (Stratagene). Quantitative RT-PCR reactions were set up in duplicate with the Power SYBR Green Master Mix (Roche) and analyzed with the Stratagene Mx3000P™ Real-Time PCR System. Comparative CT method was used to determine the relative quantification of target genes, CLEC4F (forward primer: 5′-CTTCGGGGAAGCAACAACTC-3′, reverse primer: 5′- CAAGCAACTGCACCAGAGAAC-3′) and F4/80 (forward primer: 5′-CAAGACTGACAACCAGACG-3′, reverse primer: 5′-ACAGAAGCAGAGATTATGACC-3′) normalized to a reference gene GAPDH (forward primer: 5′-GCATCCACTGGTGCTGCC-3′, reverse primer: 5′- TCATCATACTTGGCAGGTTTC-3′). Nuclease-free water was used as no-template-contained negative control.

### Western Blot Analysis

Cell and tissue samples were homogenized in RIPA buffer (50 mM Tris-HCl at pH 7.4, 150 mM NaCl, 1% NP-40, 0.5% Na-deoxycholate, and 0.1% SDS) with complete protease inhibitor cocktail (Roach). A total protein of 20∼100 µg was fractionated by 10% SDS-PAGE before transfer to nitrocellulose membrane, and subsequently probed with anti-CLEC4F mAbs (clone 3E3F9, 2 µg/ml), anti-GAPDH (Chemicon, 1∶1000), or anti-FLAG M2 Ab (Sigma-Aldrich, 1∶1000) followed by incubation with horse Radish peroxidase (HRP)-conjugated secondary antibody. Membranes were developed with Western Chemiluminescent HRP Substrate (ECL)(Millipore) and exposed to X-ray film (FUJIFILM).

### Immunohistochemistry

Formalin-fixed, paraffin-embedded tissue sections (5 µm) were deparaffinized, dehydrated, and treated with antigen retrieval buffer (0.01 M sodium citrate, pH 6.0). Sections were stained with primary antibodies: anti-CLEC4F mAb (clone 3E3F9, 2 µg/ml) and anti-F4/80 polyclonal Ab (M-300, 4 µg/ml, Santa Cruz) followed by HISTOMOUSETM-MAX kit (Zymed) according to the vendor’s instructions. Mouse IgG1 and rabbit Ig were used as isotype controls, respectively.

### Immunofluorescent Staining

Livers were perfused with PBS and fixed in 4% paraformaldehyde overnight and immersed in a serial concentration of sucrose (10% → 20% → 30%). Cryosections (5 µm) of OCT-embedded liver tissue were fixed in iced acetone for 5 min, then air-dried, followed by soaking in PBS for 5 min and incubated 3 times, for 10 min each in sodium borohydride solution (1 mg/ml in PBS). Sections were then stained with rabbit anti-F4/80 anti-sera (1∶200) and anti-CLEC4F (clone 3E3F9, 2 µg/ml), followed by FITC-conjugated goat anti-rabbit IgG and Cy3-conjugated goat-anti-mouse IgG. Nuclei were counterstained with Hoechst 33342. Mouse IgG1 and rabbit Ig were used as isotype controls, respectively. 0.3% Sudan Black in 70% EtOH were applied to slides for 10 min and then rinsed with PBS to reduce autofluorescence. After mounting, Specimens were analyzed using a confocal microscope (LSM510META; Carl Zeiss) with a narrow band pass filter. Confocal images were obtained using a 63× oil-immersion lens.

### Flow Cytometric Analysis

For cell surface staining, 1×10^5^ cells were preincubated with rat anti-mouse CD16/32 mAb (clone 2.4G2, 1 µg/ml) and mIgG (5 µg/ml) in 50 µl of FACS blocking buffer (5% (v/v) FCS, 0.1% NaN3, 5 mM EDTA in PBS) at 4°C for 10 min to prevent nonspecific binding through the Fc receptor. Cells were then incubated with PE-conjugated anti-CLEC4F (clone 3E3F9, 2 µg/ml) and Alexa Fluor 647-conjugated anti-F4/80 (AbD Serotec, 1∶50) simultaneously at 4°C for 20 min. PE-conjugated mIgG1 and Alexa Fluor 647-conjugated rat IgG2b were used as isotype controls, respectively. All samples were analyzed by FACSCalibur (BD Biosciences) using FlowJo software.

### Liposome Preparation and Kupffer Cell Depletion

Liposomes were prepared as described previously [30], [31]. In brief, 34.4 mg phosphatidylcholine and 3.2 mg cholesterol were dissolved in 4 ml chloroform in a 50 ml round-bottom flask. The thin phospholipid film was formed against the inner wall of the flask under low vacuum rotation evaporation. The phospholipid film was dispersed in 4 ml PBS or 0.6 M Cl_2_MBP by gentle rotation at room temperature to generate the PBS-encapsulated liposome and Cl_2_MBP-encapsulated liposome, respectively. The milky white suspension was kept at RT for 2 h under nitrogen (N_2_) gas for swelling. After sonication, liposomes were washed by PBS twice and collected by centrifugation. The pellets were resuspended in 1.6 ml sterilized PBS and stocked in 4°C under N_2_ gas. Kupffer cells were completely eliminated after 24 h with intravenous injection of Cl_2_MBP-encapsulated liposome (100 µl per mouse).

### Pathogens

*L. monocytogenes* were prepared as described previously [32]. In brief, *L. monocytogenes* were grown in tryptic soy broth (Difco Laboratories) at 37°C in an orbital shaker overnight and stored at −80°C with glycerol in small aliquots. The CFU concentration of the frozen infectious stocks were determined by plating a 10-fold serial dilution onto tryptic soy agar plates and used for further infection experiments.

### Direct Binding and Completive Binding of CLEC4F with α-GalCer and its Derivatives

α-GalCer was printed on the microarrays by the method reported previously [33]. Fc.CLEC4F fusion protein was diluted in the binding buffer (1% BSA, 150 mM NaCl, 2 mM CaCl_2_, 2 mM MgCl_2_, 0.05% Tween 20 and 20 mM Tris-HCl, pH7.4) and then directly applied to the sub-array of glass slides. Humidifying incubation was performed under foil for 1 h at room temperature. The slide was then washed using the following procedure: (1) washed three times with incubation buffer, (2) three times with distilled water, and (3) dried with a flow of argon gas. A solution of Cy3-labeled anti-Fc antibody was incubated on the slide for 1 h. The slide was again washed using the above procedure and the fluorescence visualized at a resolution of 5 Å m with a 595 nm laser using an ArrayWorx microarray reader (Applied Precision). For the competitive binding assay, solutions of the competitors at different concentration (5 mM to 0.1 µM) were prepared and 5 µl aliquots incubated with protein (5 µl, 25 µg/mL). An aliquot of this solution (8 µL) was loaded onto the slides and incubated for 1 h under a humidifying container at room temperature. The following procedure is the same as the direct binding assay in applying secondary antibody. To get the data, every inhibitor was assayed under this system at least 3 times to present the binding curves.

### Glycan Microarray Fabrication

Microarrays were printed (BioDot, Cartesian Technologies) onto NHS-coated glass slides by robotic pin (SMP3, TeleChem International Inc.) deposition of ∼0.7 nL amine-functionalized glycans in printing buffer (300 mM phosphate buffer, pH 8.5, 0.005% Tween-20). Each microarray slide was spotted with 50 µM of glycan, with a total of 63 glycans in an array. Printed slides were allowed to react and dry for one hour in the atmosphere of 80% humidity followed by desiccation overnight. These slides were then stored in a desiccator at room temperature before use.

### Analysis of Glycan-binding Profile

Fc.CLEC4F fusion protein (5 µg/ml) in binding buffer was applied onto glycan array slides (printed with 63 glycans, Table S1 in File S1) and incubated for 1.5 h at room temperature. After a sequential washing step with TBST, TBS, and distilled water, a secondary 40-min-incubation was done by applying DyLight 549 conjugated-goat anti-human IgG (5 µg/ml in binding buffer) onto the slides. Slides were scanned at 532 nm with ArrayWorx chip reader (Applied Precision) after washing and blow-dry. The median density of each spot was analyzed by ArrayVision (GE Healthcare), and the density of each replicate set was then calculated to give “Average Binding Intensity” and “SD (standard deviation).”

### Isolation of Kupffer Cells and Hepatic Mononuclear Cells (MNCs)

Kupffer cells were isolated by *in situ* collagenase perfusion through the portal vein as described previously [34]. Briefly, livers were perfused with 5 ml Hank's balanced salt solution (HBSS), followed by 12 ml digestion buffer (HBSS containing 0.5 mg/ml type VI collagenase (Sigma-Aldrich) and 10 µg/ml DNase I (Roach)). The livers were removed, minced and incubated in digestion buffer at 37°C for 20 min and then were pressed through a 40 µm cell strainer. Parenchymal cells were removed by low speed centrifugation at 50 *g* for 2 min. The supernatant enriched in nonparenchymal cells was centrifuged at 800 *g* for 30 min through a 25% (v/v) Percoll gradient at room temperature with no brake. The pellets of the gradient containing Kupffer cells and sinusoidal endothelial cells were washed and seeded on culture plates and incubated at 37°C for 30 min to obtain the adherent Kupffer cells. Kuppfer cells were rested for 1 day for flow cytometric analysis and further *in vitro* NKT cell activation. Hepatic mononuclear cells (MNCs) (including NKT cells) were isolated as described [35]. Briefly, liver was perfused with 5 ml ice-cold HBSS slowly to eliminate blood. Pale livers were removed, minced and incubated in RPMI medium containing 0.05% collagenase and 10 µg/ml DNase I at 37°C for 20 min and then were pressed through the cell strainer. Suspended cells were fractionated by overlaying a 33% (v/v) Percoll solution followed by centrifugation at 800 *g* for 30 min. After red blood cell lysing, the hepatic MNCs were washed and cultured at 37°C with 5% CO2 for 1 hour; non-adherent lymphocytes were obtained for further *in vitro* NKT cells activation.

### *In vitro* NKT Cells Activation

Kupffer cell (1×10^5^ cells/well) from wild-type or *Clec4f−/−* littermates were loaded with indicated concentration of α-GalCer for 3 h, then were co-cultured with hepatic MNCs (1×10^5^ cells/well) to activate NKT cells for 72 h. The supernatant was collected for detecting cytokine production. In the CD1d blocking experiment, Kupffer cells were pretreated with CD1d blocking antibody (clone 1B1, BD Biosciences) and were followed with α-GalCer loading and NKT activation.

### Cytokine Measurement by ELISA

Supernatants from α-GalCer treatment cells were collected and stored at −20°C before being used. The levels of IL-4, and IFN-γ were determined using ELISA kits (R&D System).

### Animal Model for α-GalCer-induced Hepatitis

12-wk-old wild-type or *Clec4f−/−* mice were used for animal study. α-GalCer was diluted in pyrogen-free saline to the indicated doses through intravenous injection in a total volume of 200 µl/mouse. Serum samples were collected at indicated time points for ALT and cytokine assay.

### Statistical Analysis

All data were shown as mean ±SD by using Prism software (GraphPad). An unpaired Student’s t test was used to assess for significance differences between the control groups and each treated group. A *p* value of <0.05 was regarded as significant. The survival rate was determined by Kaplan-Meier analysis with a log-rank test, and statistical significance was accepted at a *p* value of <0.05.

## Results

### CLEC4F is a Glycosylated Protein Expressed Specifically in Liver Kupffer Cells

The cDNA of murine CLEC4F (accession number: D88577) contains a 1647 bp open reading frame (ORF) and a 194 bp 3′ untranslation region (3′-UTR). The translation of the ORF predicts a polypeptide of 548 amino acids with a molecular weight of 61 kDa, which shows 80% identity to rCLEC4F. The sequence annotation of KUCR_MOUSE (P70194) revealed that the murine CLEC4F is a type-II transmembrane protein with a short amino-terminal domain (amino acid residues 1–42) in the N-terminus, followed by a hydrophobic region (amino acid residues 43–69) as transmembrane region; the third domain (amino acid residues 70–437) contains six potential N-glycosylation sites at positions 86, 92, 115, 132, 209 and 255 residues, and the fourth domain is located in the carboxyl terminus (amino acid residues 438–538) and contains a typical C-type lectin domain, also known as CRD. The CRD domain of CLEC4F displays the highest homology with hepatic asialoglycoprotein receptors (ASGP-Rs) (46.2% identity), followed by macrophage Gal/GalNAc specific lectins (MGLs) (45.7% identity), langerin (43.8% identity) and DC-SIGN (42.5% identity).

**Figure 1:**
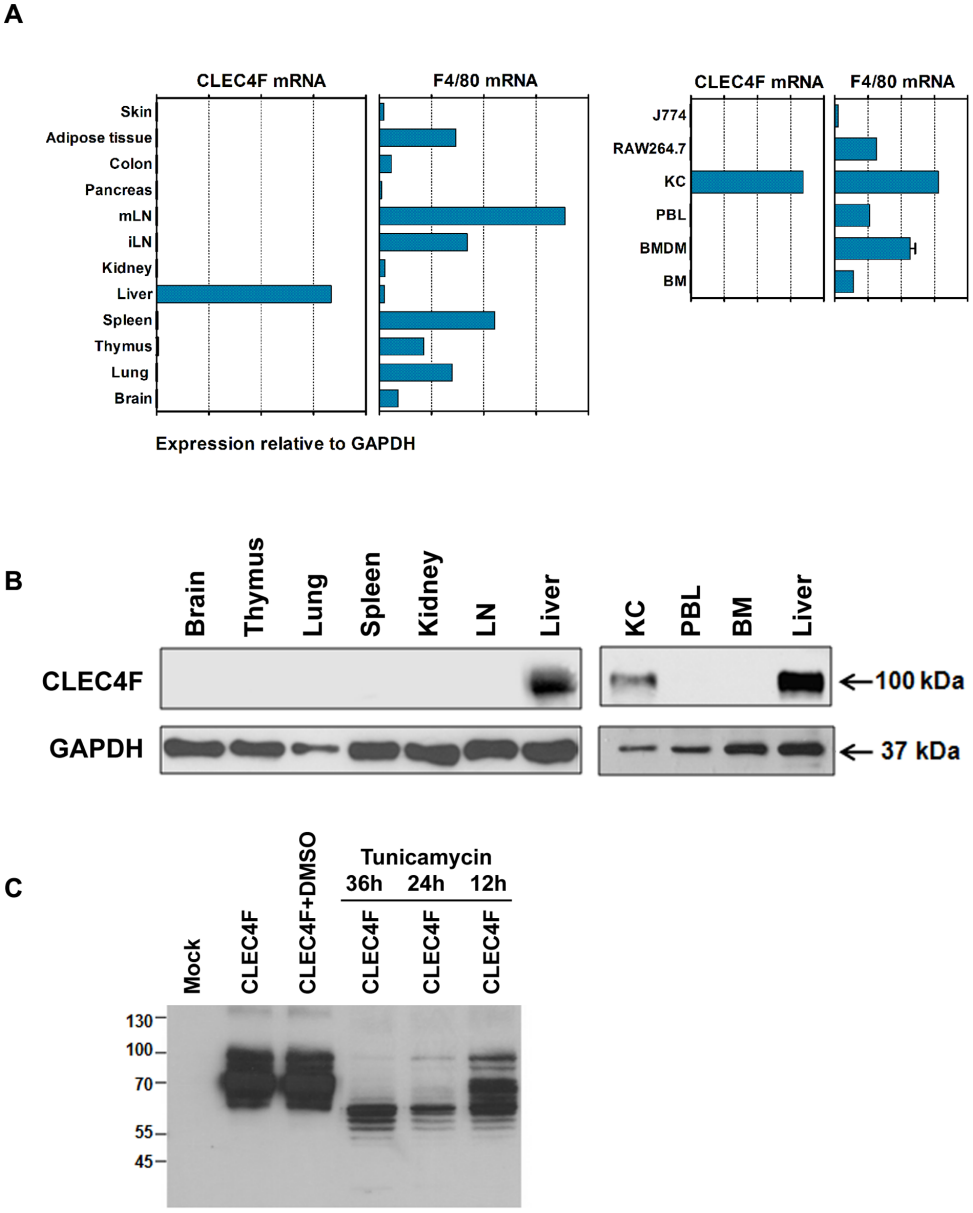
The expression and distribution of CLEC4F. (A) Tissue distribution of CLEC4F transcripts were analyzed by qRT-PCR. F4/80 is the pan macrophage marker. (B) The protein expression of CLEC4F was examined by Western blot. GAPDH was used as internal control. (C) CLEC4F is a glycoprotein. The pFLAG-CMV-2/CLEC4F-transfected 293T cells were treated with tunicamycin at 1.5 µg/ml for indicated time periods to inhibit N-linked glycosylation, and the molecular weight of CLEC4F was analyzed by Western blot. DMSO is the vehicle control. Mock indicates 293T cells transfect with pFLAG-CMV-2. mLN, mesenteric lymph node; iLN, inguinal lymph node; KC, Kupffer cells; PBL, peripheral blood leukocytes; BM, bone marrow cells; BMDM, bone marrow derived macrophages.

In contrast to F4/80, quantitative RT-PCR analysis revealed that CLEC4F mRNA is only expressed in liver and freshly isolated Kupffer cells, but not in all other tissues. In addition, CLEC4F mRNA is not detectable in bone marrow cells, bone marrow derived macrophages (BMDMs), peripheral blood cells (PBLs) or murine macrophage cell lines RAW264.7 and J774 (Figure 1A). In order to confirm CLEC4F expression at the protein level, recombinant CLEC4F was used to immunize BALB/c mice to generate anti-CLEC4F mAbs. Western blot analysis demonstrated the anti-CLEC4F mAb can recognize a 100 kDa protein in liver, which is 40 kDa more than predicted, but not the rest of tissues (left panel, Figure 1B). Moreover, CLEC4F is only detectable in fresh isolated Kupffer cells, but not in bone marrow or peripheral blood leukocytes, which contain monocytes (right panel, Figure 1B). In pFLAG-CMV-2/CLEC4F-transfected 293T cells (human embryonic kidney cells), anti-CLEC4F mAb detected polypeptides ranging from 100 kDa to 61 kDa, and addition of tunicamycin reduced the molecular weight of CLEC4F to 61 kDa (Figure 1C), indicating that CLEC4F is a glycoprotein with 40 kDa N-linked glycan, while the 70 kDa is the incompletely glycosylated form in CLEC4F-transfected 293 T cells.

### Co-expression of CLEC4F and F4/80 on Kupffer Cells

**Figure 2:**
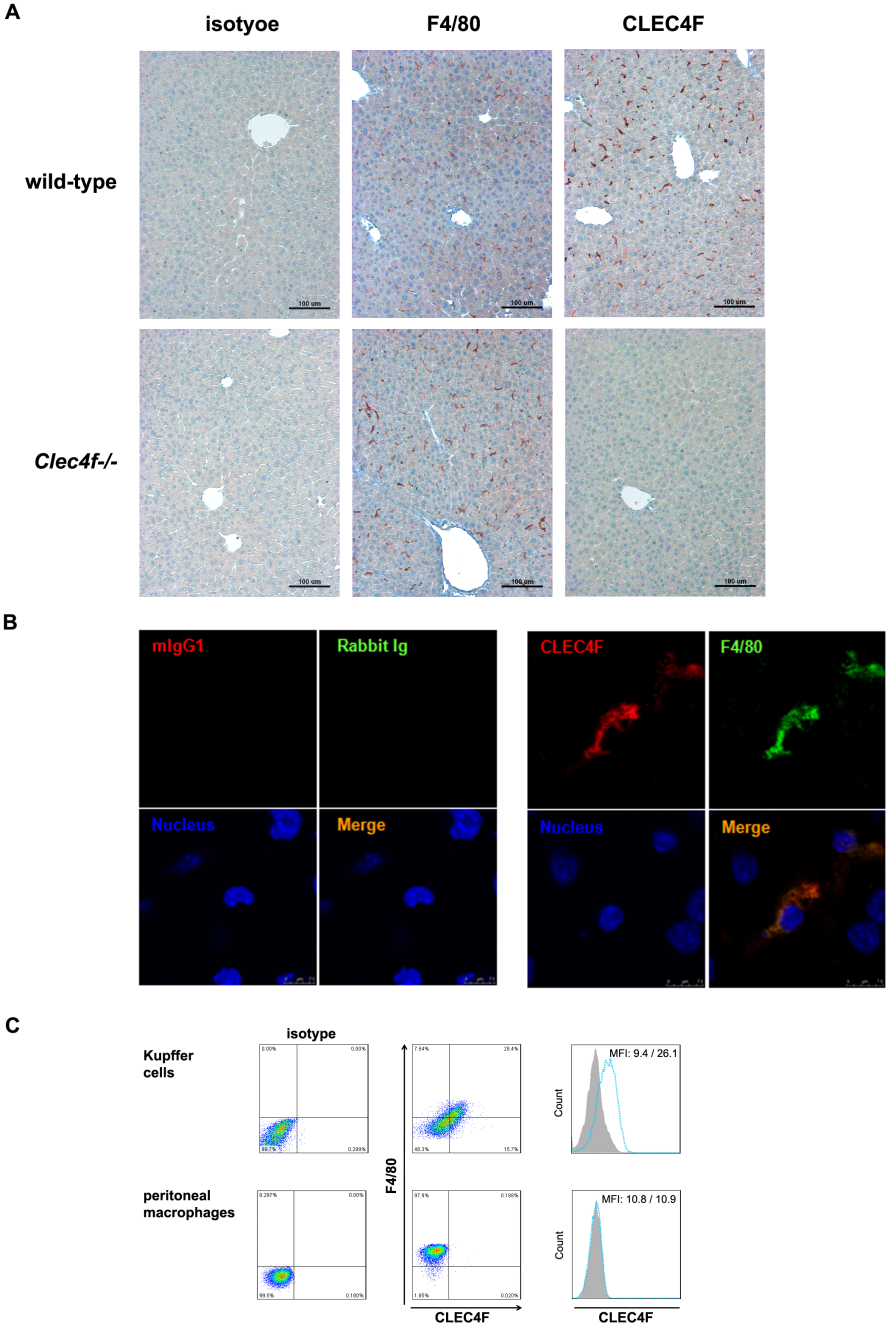
CLEC4F is co-expressed with F4/80 on liver Kupffer cells. (A) CLEC4F and F4/80 immunohistochemistry of parafilm-embedded liver sections from wild-type and *Clec4f−/−* mice. (B) Double immunofluorescence of CLEC4F and F4/80 in wild-type livers was performed. Nuclei were counterstained with Hoechst 33342. Signals were determined by confocal microscope (magnification 10×63). (C) Coexpression of CLEC4F and F4/80 on Kupffer cells, but not peritoneal macrophages. Cells were double stained with Alexa Fluor 647-conjugated anti-F4/80 and PE-conjugated anti-CLEC4F mAb. Alexa Fluor 647-conjugated rat IgG2b and PE-conjugated mIgG1 were used as isotype controls.

The localization of CLEC4F^+^ cells was determined by immunohistochemistry. By using tissue array, we found that CLEC4F is only expressed in liver sinusoid cells, but not in other tissues (Figure S2 in File S1). For further confirmation of the binding specificity of anti-CLEC4F mAb, liver sections from wild-type (upper panel, Figure 2A) and *Clec4f−/−* (lower panel, Figure 2A) mice were used. The worm-like cells located in the lumen of sinusoid were detectable by anti-CLEC4F mAb and anti-F4/80 Ab, respectively (upper panel, Figure 2A), while only anti-F4/80 Ab, but not anti-CLEC4F mAb, could detect Kupffer cells in *Clec4f−/−* liver section (lower panel, Figure 2A). In *Clec4f−/−* mice, the numbers and distribution of F4/80^+^ Kupffer cells are similar to wild-type mice, suggesting that CLEC4F is dispensable for Kupffer cell development.

Moreover, F4/80 and CLEC4F co-expressed on Kupffer cells and both signals overlapped perfectly as observed by confocal microscope (Figure 2B). Flow cytometry analysis also demonstrated that the expression of CLEC4F is appearing on the surface of Kupffer cells but not on peritoneal macrophages or other cell populations in liver or macrophage cell lines (Figure 2C & Figure S3 in File S1). These observations indicate that CLEC4F is only expressed on F4/80^+^ Kupffer cells in liver.

### Ontogeny of CLEC4F^+^ Cells

**Figure 3:**
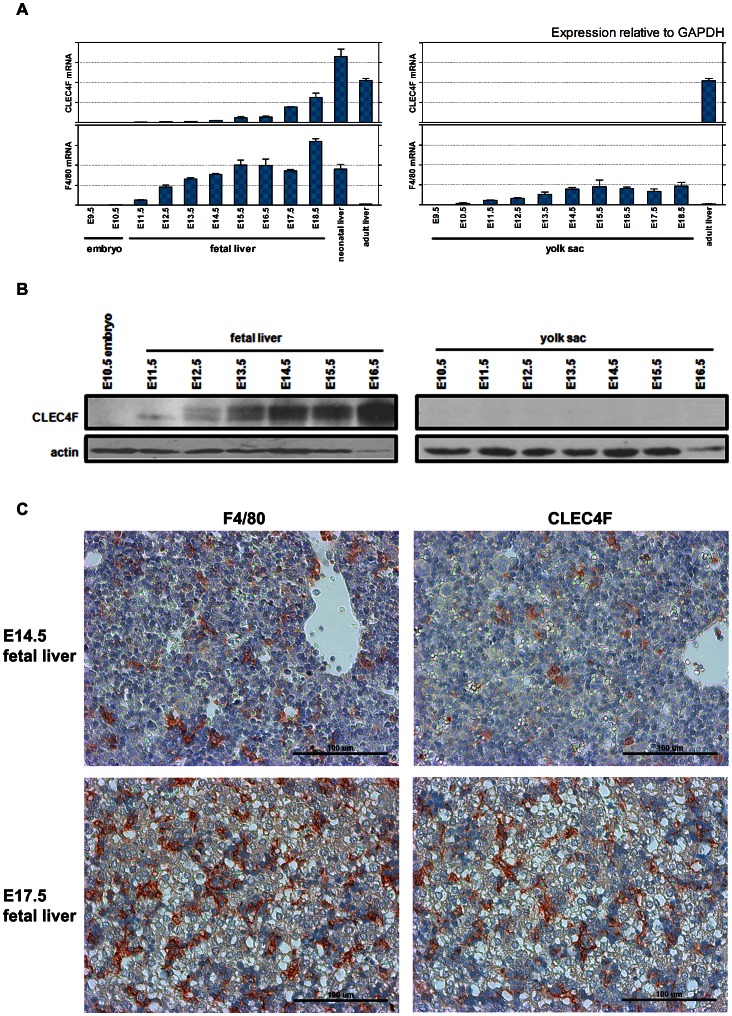
Expression of CLEC4F during embryogenesis. Yolk sac, embryo and fetal liver in various embryonic stages were collected for CLEC4F detection by (A) qRT-PCR and (B) Western blot. Actin was used as internal control. (C) F4/80 and CLEC4F immunohistochemistry of fetal liver from E14.5 and E17.5, respectively.

According to the mononuclear phagocyte system, bone marrow-derived monocytes in the blood migrate and differentiate into Kupffer cells in the liver [10], [36]. However, macrophages have been known to exist in the early stage of ontogeny long before the initiation of bone marrow hematopoiesis and to develop first in the yolk sac [37]. Since yolk sac-derived F4/80^+^ macrophages could be detected in most tissues from embryonic day 10.5 (E10.5), we wondered when and where CLEC4F^+^ cells can be detected during mouse embryogenesis. To address this question, embryos, yolk sacs and fetal livers were collected from various embryonic development stages to detect the expression of CLEC4F. We found that CLEC4F mRNA appeared in fetal liver from E11.5 and increased gradually, and reached adult levels at E18.5 (Figure 3A). Western blot analysis showed that the protein level of CLEC4F appeared in fetal liver from E12.5 (Figure 3B), but not in the yolk sac. Immunohistochemistry further showed that CLEC4F^+^ cells were present in fetal liver from both E14.5 (before the formation of bone marrow hematopoiesis) and E16.5 (Figure 3C). This observation suggests that CLEC4F is inducible in macrophages when cells migrate from yolk sac into fetal liver, and its expression is earlier than the formation of the mononuclear phagocyte system.

### Repopulation of CLEC4F^+^ and F4/80^+^ Cells after Cl_2_MBP-liposome Depletion

**Figure 4:**
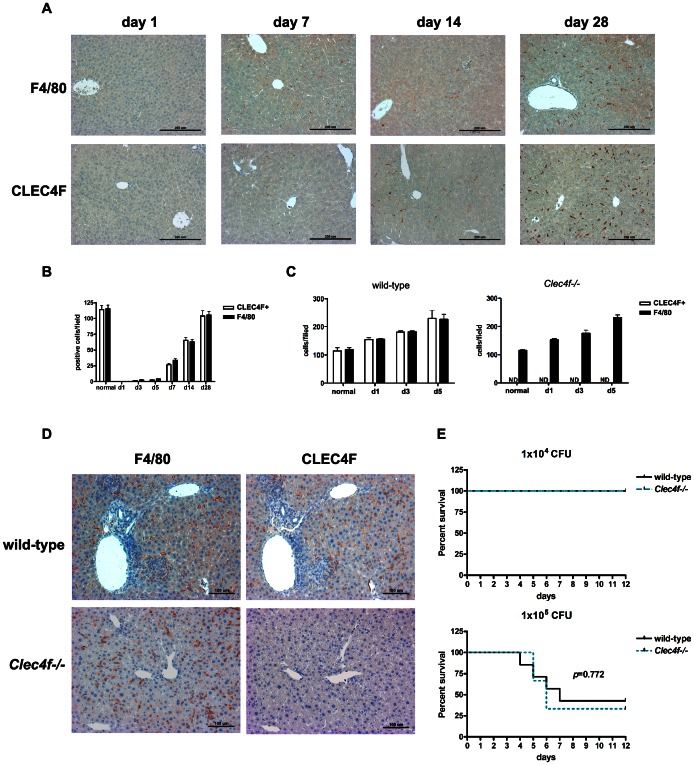
CLEC4F^+^ cells were appeared in the liver environment under Kupffer cell depletion and inflammatory stage. (A) Kupffer cells were depleted by Cl_2_MBP-encapsulated liposome by intravenous injection (100 µl/mouse) at day 0 and livers were harvest at day 1, 7, 14 and 28. F4/80 and CLEC4F immunohistochemistry of liver sections were performed. (B) The numbers of F4/80^+^ or CLEC4F^+^ cells in livers were shown. For generating inflammatory stage, wild-type and *Clec4f−/−* littermates were infected with *L. monocytogenes* (1×10^5^ CFU/mouse) intravenously. (C) The numbers of F4/80^+^ or CLEC4F^+^ cells in livers during *L. monocytogenes* infection. (D) Immunohistochemistry of *L. monocytogenes* infected livers of wild-type and *Clec4f−/−* mice at day 5 after infection. (E) Kaplan-Meier survival curves were shown for *Clec4f−/−* or wild-type littermates with *L. monocytogenes* infection. The *p* value was determined by Log-rank test.

Since CLEC4F expression is restricted to Kupffer cells, we further compared the number of CLEC4F^+^ and F4/80^+^ cells in liver under reconstitution or inflammatory stage. Firstly, we examined the amount of CLEC4F^+^ and F4/80^+^ cells during Kupffer cell repopulation after intravenous administration of liposome-encapsulated dichloromethylene diphosphonate (Cl_2_MBP) (Figure 4A & 4B). Kupffer cells could be depleted after Cl_2_MBP-encapsulated liposome treatment and will repopulate over time [16], [17], [31]. At day 1 post Cl_2_MBP-encapsulated liposome injection, Kupffer cells were depleted completely and no any F4/80^+^ or CLEC4F^+^ cells were detectable in liver (Figure 4A). At day 3 post Cl_2_MBP-encapsulated liposome injection, both CLEC4F^+^ and F4/80^+^ cells reappeared, with similar number and distribution in the liver. The numbers of CLEC4F^+^ and F4/80^+^ cells further increased and restored to original levels at day 28. Figure 4B summarized the kinetic change in the number of hepatic CLEC4F^+^ and F4/80^+^ cells after Kupffer cell depletion by Cl_2_MBP-encapsulated liposome. This observation suggests that reconstituted Kupffer cells expressed both CLEC4F and F4/80 simultaneously.

We also wondered whether the expression of CLEC4F was induced when inflammatory monocytes enter the liver environment. Liver is the most important organ in clearance of blood-borne bacteria and bacteria products, and during *L. monocytogenes* infection, inflammatory myelomonocytic cells will be recruited into the liver [38]. In order to address this question, mice were challenged with *L. monocytogenes* to generate a liver inflammatory stage to examine the population change of CLEC4F^+^ and F4/80^+^ cells during infection (Figure 4C&4D). We found that both the numbers of CLEC4F^+^ and F4/80^+^ cells were increased dramatically at day 1 post-infection, and increased at day 5 post-infection in wild-type mice (Figure 4C). The distribution and the kinetic change in the number of F4/80^+^ cells in *L. monocytogenes* infected *Clec4f−/−* mice were similar to those observed in wild-type mice (Figure 4C&4D). This observation suggests that CLEC4F is not only expressed in resident Kupffer cells, but also appeared in infiltrating F4/80^+^ mononuclear cells. Further, the clearance of blood-borne bacteria is generally attributed to Kupffer cells [39]. We also compared the susceptibility of wild-type and *Clec4f−/−* mice to *L. monocytogenes* infection to test whether CLEC4f is involved in bacterial infection. However, the survival rate of *Clec4f−/−* and wild-type littermates are similar (*p* = 0.772), suggesting CLEC4F is not critical for host defense against *L. monocytogenes* (Figure 4E).

### CLEC4F has Diverse Binding Specificity and is Involved in Glycolipid Presentation

**Figure 5:**
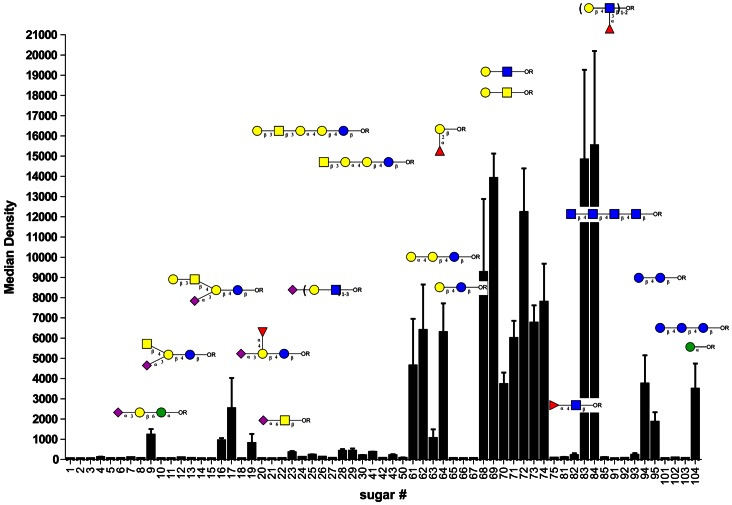
Glycan binding profile of Fc.CLEC4F. **Fc.CLEC4F (5 µg/ml) in binding buffer was applied onto glycan array slides.** The “Average Binding Intensity” and “Standard Deviation (SD)” among eight glycan replicates were analyzed by ArrayVision software (GE Healthcare).

To further explore the functions of CLEC4F, we fused the extracellular domain of CLEC4F with hIgG1 Fc portion to generate recombinant Fc.CLEC4F fusion protein, followed by probing against glycan array to understand its binding specificity. While recombinant Fc.CLEC4F fusion protein showed strong binding to the glycans with terminal Gal/GalNAc such as Gb5, Gb4, Gb3 and Gb2, it cannot bind to globo H (fucosyl Gb5). This revealed that the α1,2-linked fucose appended at the terminus of globo H was able to block the recognition of Fc.CLEC4F to terminal Gal. This phenomenon was also observed in Fc.CLEC4F binding to Lewis x and GM1, but not Lewis y epitope and fucosyl GM1 (both Lewis y and fucosyl GM1 bearing α1,2-linked fucose in the terminal). (Figure 5 & Table S1 in File S1). Thus, fucosylation has profound inhibitory effect on CLEC4F binding to glycans.

**Figure 6:**
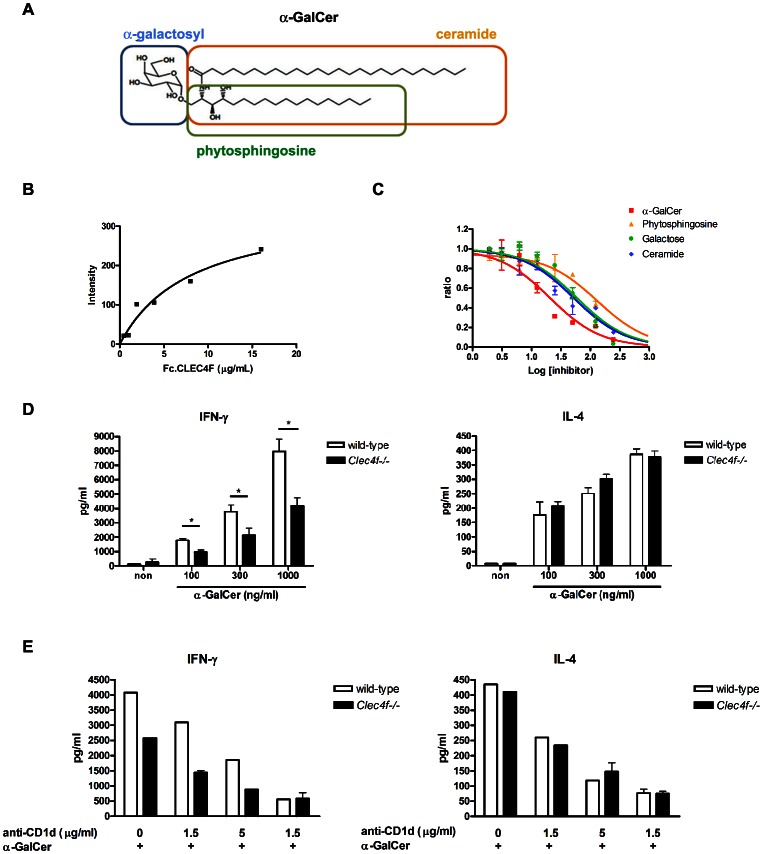
CLEC4F is involved in the presentation of α-GalCer to NKT cells. (A) The schematic structure of α-GalCer. (B) The binding curves were obtained from the function of Fc.CLEC4F concentration and fluorescence intensity determined from array images. (C) Competition experiment between solution and surface for Fc.CLEC4F binding to α-GalCer and three derivatives. At different concentration of the competitors, binding curves were obtained from the bound Fc.CLEC4F concentration and fluorescence. (D) Secretion of IFN-γ and IL-4 of NKT cells after incubation of α-GalCer presented by Kupffer cells isolated from wild-type and *Clec4f−/−* mice. Kupffer cells (1×10^5^ cells) isolated form wild-type and *Clec4f−/−* littermates were treated with serial concentration of α-GalCer (10, 30, 100, 300 and 1000 ng/ml) then co-cultured with NKT cells (1×10^5^ cells). The supernatant were collected at 72 h post-stimulation and detected the IL-4 and IFN-γ production by ELISA. (E) Effect of CD1d-blocking antibody in the α-GalCer presentation by wild-type and *Clec4f−/−* Kupffer cells. Kupffer cells were pretreated with CD1d blocking antibody. Three hours later, α-GalCer (300 ng/ml) was added and co-cultured with NKT cells. The supernatant were collected at 72 h post-stimulation and detected the IL-4 and IFN-γ production by ELISA.

**Figure 7:**
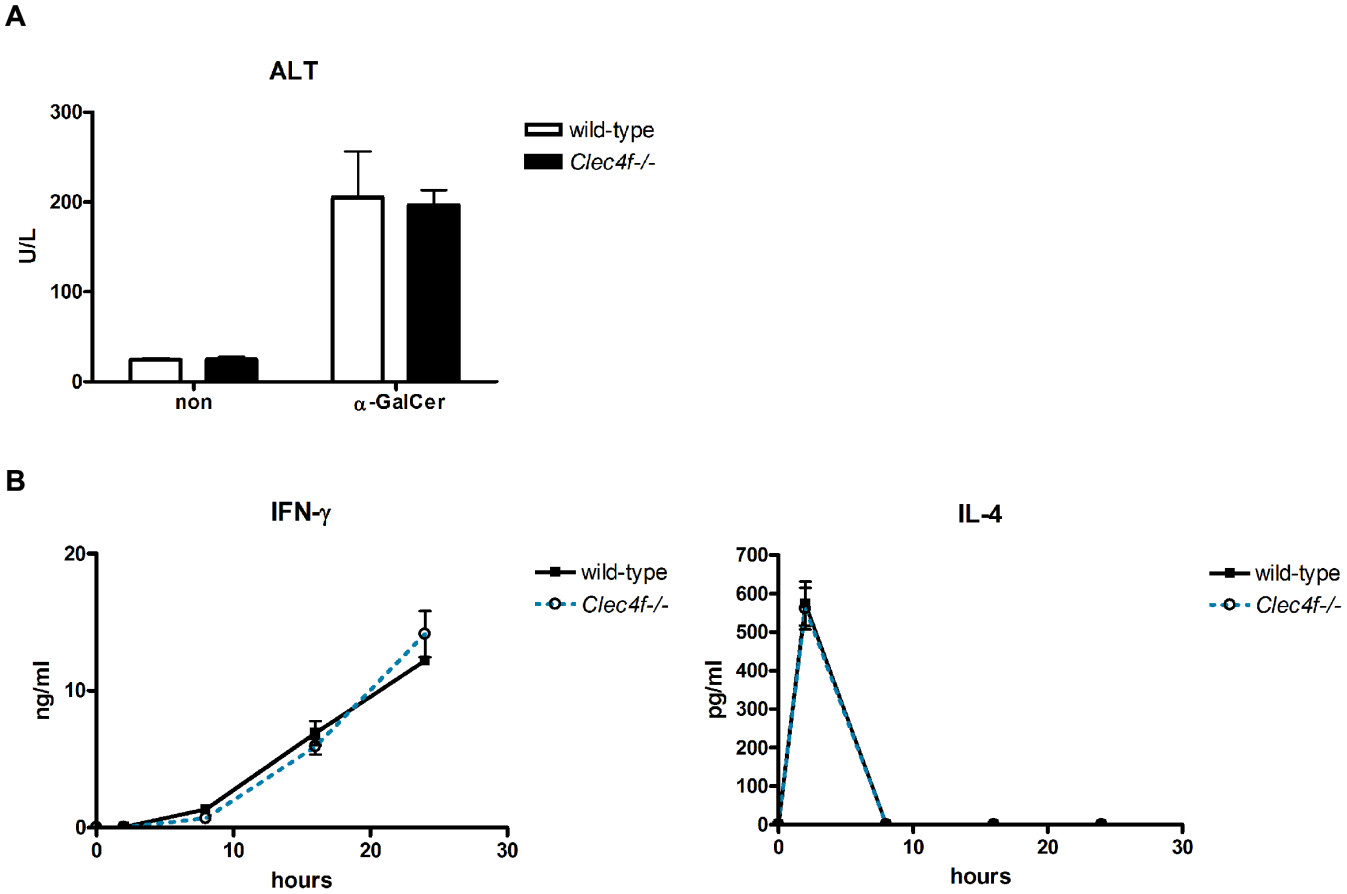
*Clec4f−/−* mice showed a similar response of α-GalCer induced activation to wild-type mice *in vivo*. Both wild-type and *Clec4f−/−* littermates were treated with α-GalCer (1 µg/mouse) intravenously. Serum levels of (A) ALT and (B) cytokines, including IL-4 and IFN-γ were analyzed for samples taken at indicated time points after α-GalCer challenge. Data are representative of three independent experiments with similar results. (3 mice in each group).

Based on its strong binding to Gal, we asked whether CLEC4F is able to interact with α-GalCer (Figure 6A), which is a glycolipid with Gal terminus and is a potent immunomodulator in the activation of NKT cells [22], [33], [40]. We firstly asked whether CLEC4F interacts with α-GalCer and plays a role in the presentation to NKT cells. To address this question, recombinant Fc.CLEC4F was incubated with surface-bound α-GalCer on microarray. The binding affinity of Fc.CLEC4F with α-GalCer was determined to be 100 nM (dissociation constant), indicating a multivalent interaction of Fc.CLEC4F with α-GalCer (Figure 6B). In a competitive binding experiment, α-GalCer and three truncated derivatives (phytosphingosine, Gal, and ceramide) in solution competed with immobilized α-GalCer for the binding sites on the CLEC4F (Figure 6C). The binding strength was in an order of α-GalCer>ceramide ≈ Gal >phytoshpingosine. This observation indicates that both Gal and ceramide were involved in Fc.CLEC4F and α-GalCer interactions. We further asked whether CLEC4F is involved in α-GalCer presentation to hepatic NKT cells. To address this question, Kupffer cells isolated from *Clec4f−/−* or wild-type littermates were incubated with α-GalCer, followed by incubation with hepatic lymphocytes (including hepatic NKT cells). We were surprised to find that the secretion of IFN-γ is downregulated in hepatic lymphocytes stimulated with α-GalCer-loaded *Clec4f−/−* Kupffer cells, while the production of IL-4 is similar under the same conditions (Figure 6D). Interestingly, blocking antibody against CD1d further suppressed the secretion of IFN-γ in a dose-dependent manner, and there is no difference for IFN-γ and IL-4 secretion between *Clec4f−/−* and wild-type Kupffer cells in the presence of high dose (15 µg/ml) anti-CD1d mAb (Figure 6E). However, serum levels of ALT, IFN-γ and IL-4 are similar between *Clec4f−/−* and wild-type littermates (Figure 7), suggesting dendritic cells and other antigen presenting cells in liver can also present α-GalCer efficiently to NKT cells even in the presence of CLEC4F.

## Discussion

Kupffer cells are the largest and heterogeneous population of resident macrophages in the liver [9]. The biological function of Kupffer cells have been implicated in the pathogenesis of host defense, bilirubin metabolism, ischemia-reperfusion injury, viral hepatitis, steatohepatitis, alcoholic liver disease, intrahepatic cholestasis, activation or rejection of the liver during liver transplantation, and liver fibrosis, as well as non-alcoholic fatty liver disease [41]. In this study, we demonstrated clearly that the expression of CLECF4F is restricted on Kupffer cells, and facilitates α-GalCer presentation to NKT cells. In addition, CLEC4F displays diverse binding specificity to glycans. Thus, CLEC4F is not only a Kupffer cell-specific marker, but may also contribute to the presentation of other glycans and glycolipids to activate NKT cells.

In contrast to F4/80, which is a constitutively expressed surface marker for both peripheral and resident macrophages [12], [13], [42], CLEC4F is inducible in the liver microenvironment. The first evidence comes from the observation that CLEC4F is detectable in fetal liver from E11.5, but not in yolk sac (Figure 3) during embryogenesis. Yolk sac is the first organ where the macrophages develop. It has been demonstrated that macrophages occur in the yolk sac of animal and human embryos. The F4/80^+^ primitive macrophages appear in murine yolk sac at E9, and then differentiate into fetal macrophages [36], [37], [42], [43], [44]. Fetal liver begins to form its fundamental structure at E10, and primitive macrophages are detected in the hepatic sinusoid [36]. Our findings suggest that the expression of CLEC4F appeared when primitive macrophages migrated into fetal liver. The second evidence comes from the observation that both the number of F4/80^+^ and CLEC4F^+^ cells in the liver were increased and similar during *L. monocytogenes* infection. (Figure 4D). This suggests the infiltrating F4/80^+^ cells become CLEC4F^+^ upon entry to the liver. Finally, we found the expression of CLEC4F is lost when Kupffer cells were cultured *in vitro* for 7 days (Figure S4 in File S1). In addition, CLEC4F is not expressed on the murine Kupffer cell line, KC13-2 [8] (Figure S3 in File S1), suggesting Kupffer cells may fail to maintain CLEC4F expression under culture condition. It would be interesting to understand what kinds of signals or molecules are necessary to regulate CLEC4F expression in the liver microenvironment in the future.

Several C-type lectin receptors, including Dectin-1, Dectin-2, DC-SIGN, and Mincle have been shown to recognize cell wall components of *Candida albicans* (*C. albicans*) [46], [49], [50]. We also found that CLEC4F is able to interact with polysaccharides isolated from *C. albicans*, but the survival rates of *Clec4f−/−* mice is similar to wild-type littermates after intravenous injection of *C. albicans* (Figure S5A in File S1), even thought *Clec4f−/−* Kupffer cells were shown to be unresponsive to *C. albicans* compare to wild-type Kupffer cells *in vitro* (Figure S5B in File S1). Furthermore, there is no difference in histopathological change between *Clec4f−/−* and wild-type littermates (Figure S5C in File S1), suggesting CLEC4F is dispensable in host defense against candida invasion. Further study is necessary to reveal the functions of CLEC4F in Kupffer cells-mediated immunomodulation in the future.

## Supporting Information

File S1 **Figure S1, S2, S3, S4, S5 and Table S1.** Figure S1 in File S1. Generation of Clec4f knockout mice. (A) Clec4f targeting strategy. Genomic structure of the wild-type and targeted alleles of Clec4f gene was destroyed by inserting the EGFP gene into the exon 4 of Clec4f gene. (B) Southern blot hybridization of genomic DNA from wild-type (+/+), heterozygous (+/−) and homozygous (−/−) offspring by using 3′-flanking probe. (C) Western blot analysis of CLEC4F protein expression from liver extracts. B: BamHI; E: EcoRI; EV: EcoRV; H: HindIII. Figure S2 in File S1. Characterization of CLEC4F expression by murine tissue array screening. Immunohistochemistry of CLEC4F (B, D, F, H, J, L, N, P, R) in mouse tissues, including liver (A, B), BM (C, D), thymus (E,F), spleen (G, H), LN (I, J), brain (K, L), lung (M, N), kidney (O, P) and gut (G, R) and mIgG1 was used as isotype control (A, C, E, G, I, K, M, O, Q). Sections were counterstained with hematoxylin. BM, bone marrow; LN, lymph node; M, medulla; C, cortex; W, while pulp; R, red pulp; F, follicle. Scale bar: 100 µm. Figure S3 in File S1. CLEC4F expression screening. (A) CLEC4F-transfected 293T cells, (B) macrophage cell lines, RAW264.7 and J774, and Kupffer cell line, KC13-2, and (C) hepatic cell populations were used for CLEC4F staining. Cells were stained with lineage markers: F4/80, NK1.1, B220 or TCR, and double or triple stained with CLEC4F. Mouse IgG1 was used as isotype control. Signals were determined by FACS Calibur for flow cytometric analysis. Figure S4 in File S1. The expression of CLEC4F is decreased under culture condition. Kupffer cells isolated from C57BL/6 mice were cultured in RPMI 1640 supplemented with 10% (v/v) heat-inactivated fetal bovine serum at 37°C in 5% CO2. The expression of CLEC4F was detected by Western blot in indicated culture time periods. Figure S5 in File S1. CLEC4F is dispensable for C. albicans infection in vivo. (A) The survival rate of wild-type and Clec4f−/− littermates after C. albicans infection. The p value was determined by using Kaplan-Meier survival analysis and compared by Log-rank test. (B) Kupffer cells (1×105 cells) isolated from wild-type and Clec4f−/− littermates were stimulated with heat-killed conidia (HK C), heat-killed hyphae (HK H) and live conidida (L C) in 1×106 CFU/ml. The supernatant were collected at 24 h post-stimulation and detected the level of IL-6 and TNF-α by ELISA. *: p<0.05; **: p<0.01; ***: p<0.005. “n.d.” indicates non-detectable. (C) The liver and kidney sections from C. albicans infected mice were analyzed by H&E staining. Representative histopathological sections of liver and kidney from mice infected with 1×106 CFU of C. albicans. Table S1 in File S1. Glycan binding profile of Fc.CLEC4F. Gal: galactose, GalNAc: N-acetylgalactosamine, Glc: glucose, GlcNAc: N-acetylglucosamine, Man: mannose, Fuc: fucose, Neu5Ac: N-acetylneuraminic acid, Neu5Gc: N-glycolylneuraminic acid.
(PDF)
