# Supplementary material for: CLEC4F Is an Inducible C-Type Lectin in F4/80-Positive Cells and Is Involved in Alpha-Galactosylceramide Presentation in Liver
Source: PLoS One. 2013 Jun 6;8(6):e65070. doi: 10.1371/journal.pone.0065070 (PMC3675125; doi:10.1371/journal.pone.0065070)
Supplement: File S1 — Figure S1, S2, S3, S4, S5 and Table S1. Figure S1 in File S1. Generation of Clec4f knockout mice. (A) Clec4f targeting strategy. Genomic structure of the wild-type and targeted alleles of Clec4f gene was destroyed by inserting the EGFP gene into the exon 4 of Clec4f gene. (B) Southern blot hybridization of genomic DNA from wild-type (+/+), heterozygous (+/−) and homozygous (−/−) offspring by using 3′-flanking probe. (C) Western blot analysis of CLEC4F protein expression from liver extracts. B: BamHI; E: EcoRI; EV: EcoRV; H: HindIII. Figure S2 in File S1. Characterization of CLEC4F expression by murine tissue array screening. Immunohistochemistry of CLEC4F (B, D, F, H, J, L, N, P, R) in mouse tissues, including liver (A, B), BM (C, D), thymus (E,F), spleen (G, H), LN (I, J), brain (K, L), lung (M, N), kidney (O, P) and gut (G, R) and mIgG1 was used as isotype control (A, C, E, G, I, K, M, O, Q). Sections were counterstained with hematoxylin. BM, bone marrow; LN, lymph node; M, medulla; C, cortex; W, while pulp; R, red pulp; F, follicle. Scale bar: 100 µm. Figure S3 in File S1. CLEC4F expression screening. (A) CLEC4F-transfected 293T cells, (B) macrophage cell lines, RAW264.7 and J774, and Kupffer cell line, KC13-2, and (C) hepatic cell populations were used for CLEC4F staining. Cells were stained with lineage markers: F4/80, NK1.1, B220 or TCR, and double or triple stained with CLEC4F. Mouse IgG1 was used as isotype control. Signals were determined by FACS Calibur for flow cytometric analysis. Figure S4 in File S1. The expression of CLEC4F is decreased under culture condition. Kupffer cells isolated from C57BL/6 mice were cultured in RPMI 1640 supplemented with 10% (v/v) heat-inactivated fetal bovine serum at 37°C in 5% CO2. The expression of CLEC4F was detected by Western blot in indicated culture time periods. Figure S5 in File S1. CLEC4F is dispensable for C. albicans infection in vivo. (A) The survival rate of wild-type and Clec4f−/− littermates after C. albicans [file pone.0065070.s001.pdf]

SUPPORTING INFORMATION LEGENDS

Figure S1 in File S1. Generation of *Clec4f* knockout mice. (A) *Clec4f* targeting strategy. Genomic structure of the wild-type and targeted alleles of *Clec4f* gene was destroyed by inserting the *EGFP* gene into the exon 4 of *Clec4f* gene. (B) Southern blot hybridization of genomic DNA from wild-type (+/+), heterozygous (+/-) and homozygous (-/-) offspring by using 3'-flanking probe. (C) Western blot analysis of CLEC4F protein expression from liver extracts. B: BamHI; E: EcoRI; EV: EcoRV; H: HindIII.

Figure S2 in File S1. Characterization of CLEC4F expression by murine tissue array screening. Immunohistochemistry of CLEC4F (B, D, F, H, J, L, N, P, R) in mouse tissues, including liver (A, B), BM (C, D), thymus (E,F), spleen (G, H), LN (I, J), brain (K, L), lung (M, N), kidney (O, P) and gut (G, R) and mIgG1 was used as isotype control (A, C, E, G, I, K, M, O, Q). Sections were counterstained with hematoxylin. BM, bone marrow; LN, lymph node; M, medulla; C, cortex; W, while pulp; R, red pulp; F, follicle. Scale bar: 100 µm.

Figure S3 in File S1. CLEC4F expression screening. (A) CLEC4F-transfected 293T cells, (B) macrophage cell lines, RAW264.7 and J774, and Kupffer cell line, KC13-2, and (C) hepatic cell populations were used for CLEC4F staining. Cells were stained with lineage markers: F4/80, NK1.1, B220 or TCR, and double or triple stained with CLEC4F. Mouse IgG1 was used as isotype control. Signals were determined by FACS Calibur for flow cytometric analysis.

Figure S4 in File S1. The expression of CLEC4F is decreased under culture condition. Kupffer cells isolated from C57BL/6 mice were cultured in RPMI 1640 supplemented with

10 % (v/v) heat-inactivated fetal bovine serum at 37℃ in 5% CO2. The expression of CLEC4F was detected by Western blot in indicated culture time periods.

Figure S5 in File S1. CLEC4F is dispensable for *C. albicans* infection *in vivo*. (A) The survival rate of wild-type and *Clec4f-/-* littermates after *C. albicans* infection. The *p* value was determined by using Kaplan-Meier survival analysis and compared by Log-rank test. (B) Kupffer cells ($1x10^5$ cells) isolated from wild-type and *Clec4f-/-* littermates were stimulated with heat-killed conidia (HK C), heat-killed hyphae (HK H) and live conidida (L C) in $1x10^6$ CFU/ml. The supernatant were collected at 24 h post-stimulation and detected the level of IL-6 and TNF-α by ELISA. *: $p<0.05$; **: $p<0.01$; ***: $p<0.005$. “n.d.” indicates non-detectable. (C) The liver and kidney sections from *C. albicans* infected mice were analyzed by H&E staining. Representative histopathological sections of liver and kidney from mice infected with $1x10^6$ CFU of *C. albicans*.

Table S1 in File S1. Glycan binding profile of Fc.CLEC4F. Gal: galactose, GalNAc: N-acetylgalactosamine, Glc: glucose, GlcNAc: N-acetylglucosamine, Man: mannose, Fuc: fucose, Neu5Ac: N-acetylneuraminic acid, Neu5Gc: N-glycolylneuraminic acid.

Figure S1

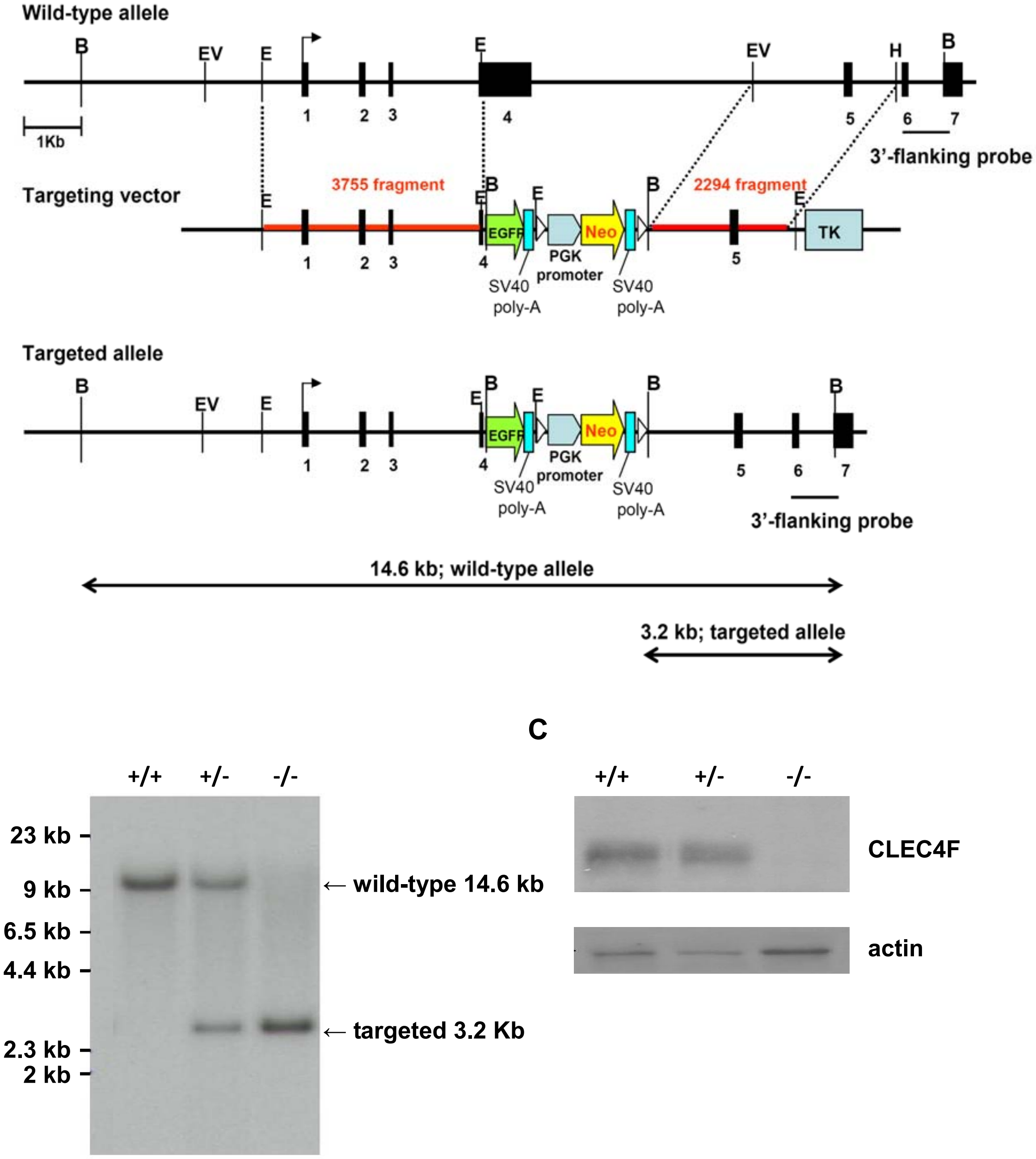
Wild-type allele
B
EV
E
E
EV
H
B
1
2
3
4
5
6
7
1Kb
3'-flanking probe
Targeting vector
3755 fragment
2294 fragment
E
E
B
E
B
E
EGFP
Neo
TK
PGK promoter
SV40 poly-A
SV40 poly-A
Targeted allele
14.6 kb; wild-type allele
3.2 kb; targeted allele
B
+/+
+/-
-/-
23 kb
9 kb
6.5 kb
4.4 kb
2.3 kb
2 kb
← wild-type 14.6 kb
← targeted 3.2 Kb
C
CLEC4F
actin

Figure S2

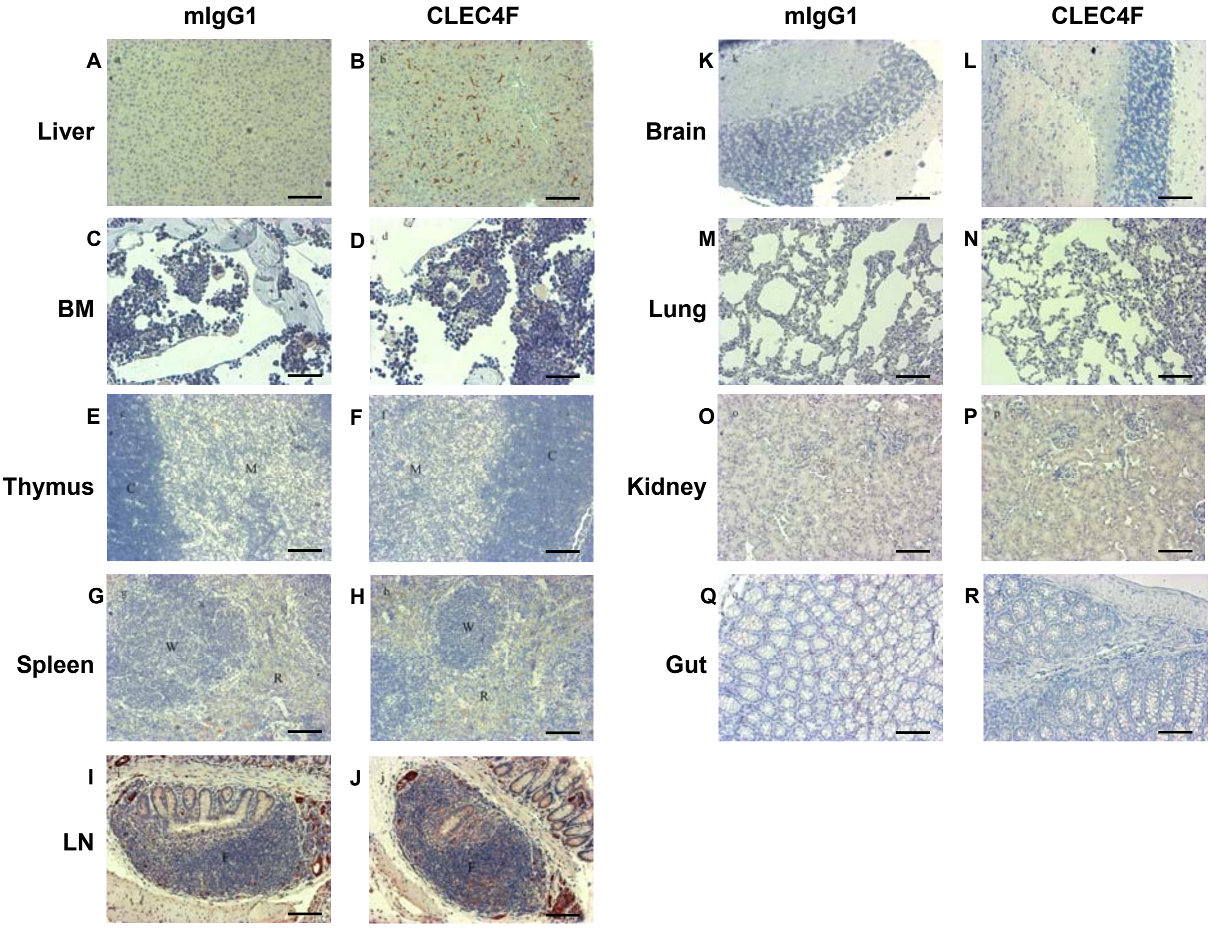

mIgG1
CLEC4F
mIgG1
CLEC4F
A
B
K
L
Liver
Brain
C
D
M
N
BM
Lung
E
F
O
P
Thymus
Kidney
G
H
Q
R
Spleen
Gut
I
J
LN

Figure S3

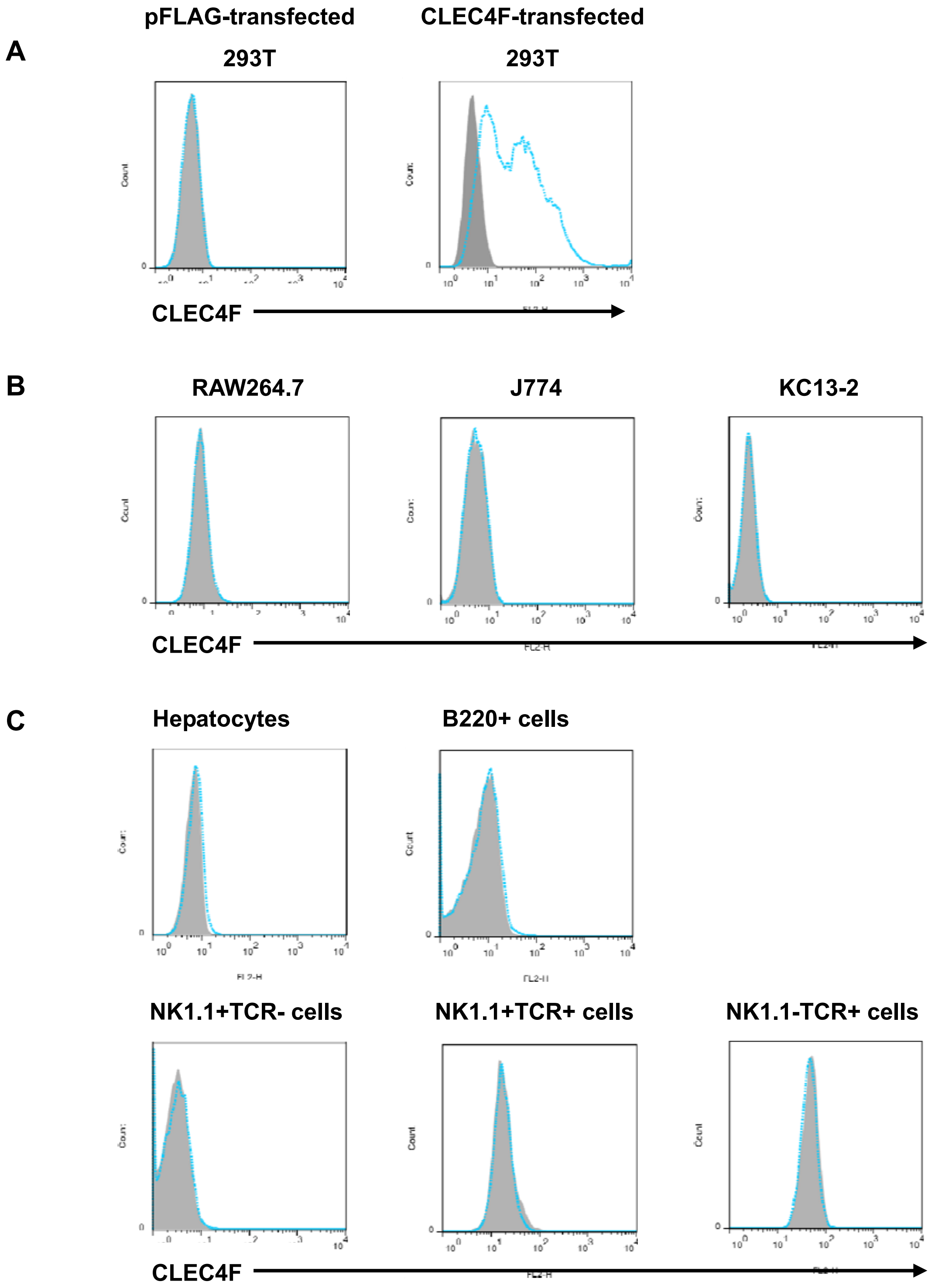
A
pFLAG-transfected
293T
CLEC4F-transfected
293T
CLEC4F
B
RAW264.7
J774
KC13-2
CLEC4F
C
Hepatocytes
B220+ cells
NK1.1+TCR- cells
NK1.1+TCR+ cells
NK1.1-TCR+ cells
CLEC4F

Figure S4

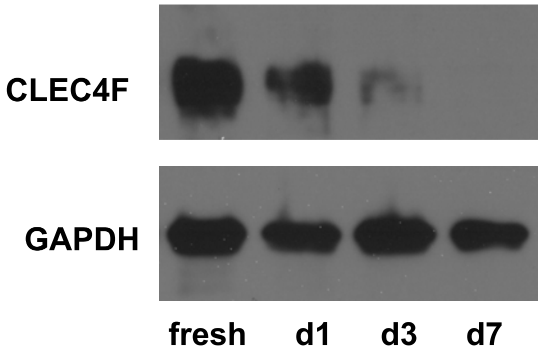

Figure S5

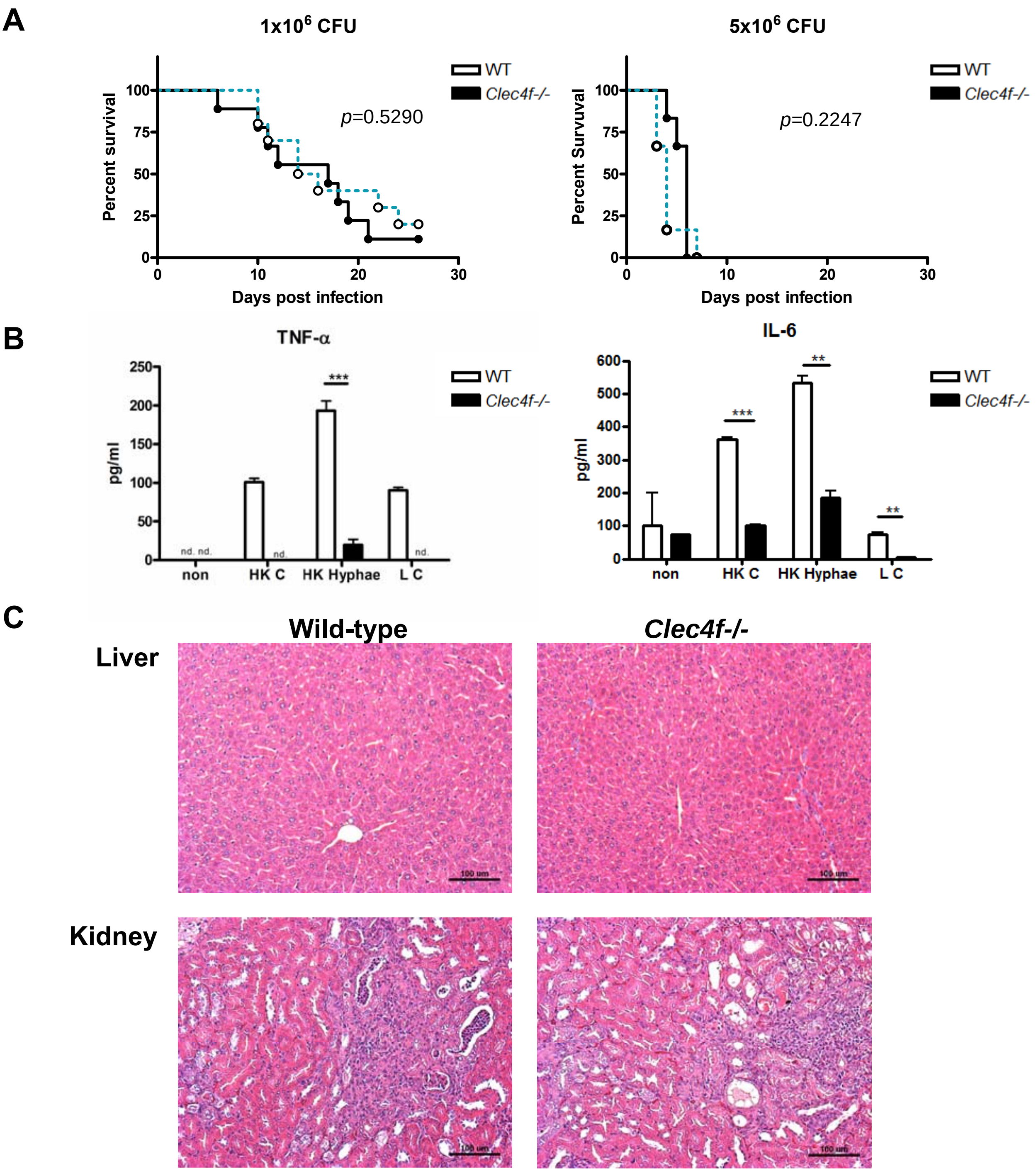
A
1x10^6 CFU
Percent survival
100
75
50
25
0
0
10
20
30
Days post infection
WT
Clec4f-/-
p=0.5290
5x10^6 CFU
Percent Survival
Days post infection
p=0.2247
B
TNF-α
pg/ml
250
200
150
100
50
0
nd. nd.
nd.
nd.
***
non
HK C
HK Hyphae
L C
IL-6
600
500
400
300
200
100
0
***
**
**
C
Wild-type
Clec4f-/-
Liver
Kidney

Table S1

Sugar No.	Glycan*/Trivial Name	Symbolic Representation	Average Binding Intensity	SD
1	Neu5Acα3Gal	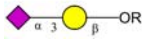	45.00	2.27
2	GM3	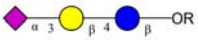	42.39	1.90
3	sLacNAc	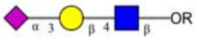	41.44	1.05
4	sLacNAc6SO_3	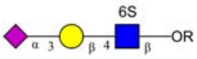	99.50	42.61
5	sLe^x epitope	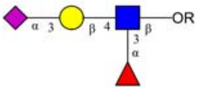	51.88	5.01
6	Neu5Acα3Galβ3GlcNAc	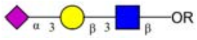	53.81	6.39
7	Neu5Acα3Galβ3GlcNAc 6SO_3	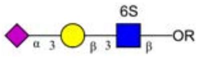	95.31	22.09
8	α3Neu5Ac on Core 1	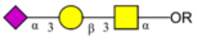	57.19	5.68
9	Neu5Acα3Galβ6Man	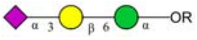	1214.44	284.99
10	SSEA4G epitope α	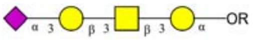	47.75	5.90
11	SSEA4G epitope β	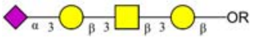	46.94	3.14
12	SSEA4	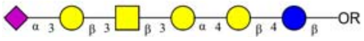	86.69	11.40
13	Neu5Acα3Galβ3(Neu5Ac α3Galβ6)GlcNAc	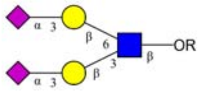	60.31	11.09
14	Neu5Acα3Galβ3(Neu5Ac α3Galβ6)Man	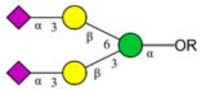	42.00	1.34
15	DSGG epitope	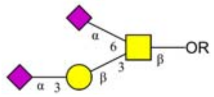	43.94	2.34
16	GM2	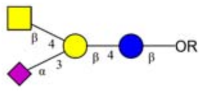	928.50	126.41
17	GM1	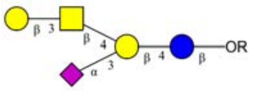	2520.94	1504.74
18	Fucosyl GM1	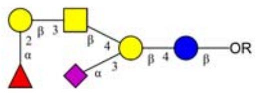	43.50	0.93

19	Neu5Acα3(Fucα4)Galβ4 Glc	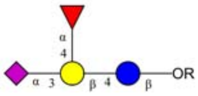	798.75	466.21
20	Neu5Acα8Neu5Ac	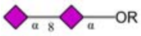	51.94	5.85
21	Neu5Ac	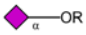	45.13	1.73
22	Neu5Acα6Gal	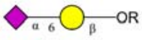	54.38	6.09
23	sTn	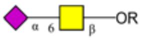	337.22	82.89
24	6’sLacNAc	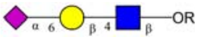	116.44	16.98
25	6’sLac	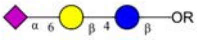	221.28	30.45
26	6’sTF	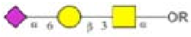	121.44	17.28
27	Neu5Acα6Galβ3GlcNAc	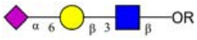	68.39	6.86
28	6’sLacNAc6SO_3	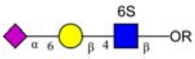	407.33	106.08
29	6’sLacNAc-LacNAc	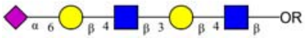	418.61	126.22
30	6’sLacNAc-$(LacNAc)_2$	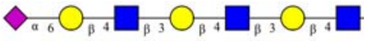	188.88	36.43
41	Neu5Gc	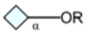	358.94	28.89
42	Neu5Gcα3Gal	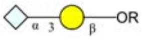	66.44	15.34
43	Neu5Gcα6Gal	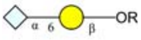	193.06	63.67
50	Neu5Acα8-SSEA4	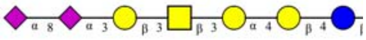	70.88	30.67
61	Gb5	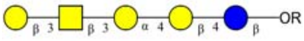	4634.06	2320.13
62	Gb4	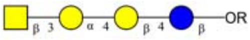	6391.94	2257.84
63	Gb3	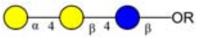	1048.94	437.99
64	Gb2	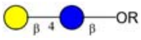	6283.06	1435.70
65	Globo H	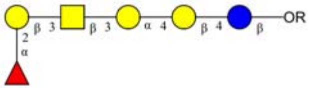	58.50	8.66
66	Bb4	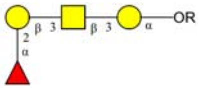	46.94	3.34

67	Bb3	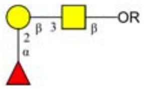	54.94	11.07
68	Bb2	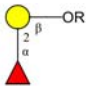	9254.00	3623.35
69	TF	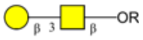	13903.44	1224.28
70	Galβ3GalNAcβ3Gal	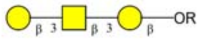	3711.75	586.25
71	LacNAc	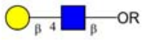	5990.81	869.42
72	LacNAc6SO_3	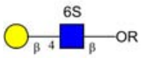	12223.75	2169.64
73	Tn	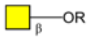	6745.50	877.61
74	Galβ3GlcNAc	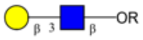	7784.94	1894.58
75	Le^y epitope	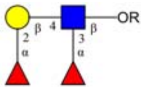	75.75	14.88
81	Fucα3GlcNAc	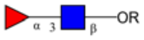	96.13	30.71
82	Fucα4GlcNAc	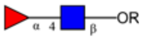	197.25	107.80
83	Le^x epitope	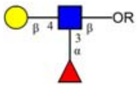	14816.13	4455.13
84	Le^x dimer epitope	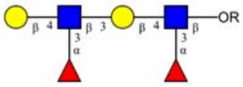	15518.19	4675.13
85	KH-1 epitope	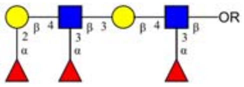	88.44	30.72
91	Chitobiose	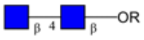	45.19	3.23
92	Chitotriose	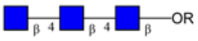	67.81	13.50
93	Chitotetraose	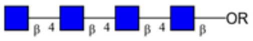	212.88	101.53
94	Cellobiose	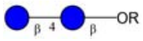	3740.75	1408.06
95	Cellotriose	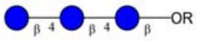	1850.50	486.76
101	Man4	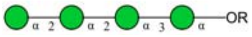	50.69	6.86
102	Man7	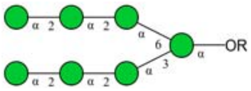	81.13	15.49

103	Man8	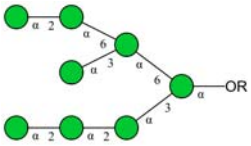	63.00	17.35
104	Man1	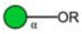	3485.19	1261.09
